# Withaferin A and Ovarian Cancer Antagonistically Regulate Skeletal Muscle Mass

**DOI:** 10.3389/fcell.2021.636498

**Published:** 2021-02-25

**Authors:** Alex R. Straughn, Natia Q. Kelm, Sham S. Kakar

**Affiliations:** ^1^James Graham Brown Cancer Center, University of Louisville, Louisville, KY, United States; ^2^Department of Physiology, University of Louisville, Louisville, KY, United States

**Keywords:** atrophy, cachexia, satellite cells, catabolism, ovary

## Abstract

Cachexia is a complex wasting syndrome that overwhelmingly affects the majority of late-stage cancer patients. Additionally, there are currently no efficacious therapeutic agents to treat the muscle atrophy induced by the cancer. While several preclinical studies have investigated the molecular signals orchestrating cachexia, very little information exists pertaining to ovarian cancer and the associated cachexia. Work from our lab has recently demonstrated that the steroidal lactone Withaferin A (WFA) is capable of attenuating the atrophying effects of ovarian cancer in a preclinical mouse model. However, it remained to be determined whether WFA’s effect was in response to its anti-tumorigenic properties, or if it was capable of targeting skeletal muscle directly. The purpose of this study was to uncover whether WFA was capable of regulating muscle mass under tumor-free and tumor-bearing conditions. Treatment with WFA led to an improvement in functional muscle strength and mass under tumor-bearing and naïve conditions. WFA and ovarian cancer were observed to act antagonistically upon critical skeletal muscle regulatory systems, notably myogenic progenitors and proteolytic degradation pathways. Our results demonstrated for the first time that, while WFA has anti-tumorigenic properties, it also exerts hypertrophying effects on skeletal muscle mass, suggesting that it could be an anti-cachectic agent in the settings of ovarian cancer.

## Introduction

Late-stage cancer patients frequently exhibit the complex metabolic syndrome cachexia (Gadducci et al., 2001). Cachexia is primarily marked by a loss of muscle strength and mass (Fearon et al., 2011; Fearon et al., 2012). This sequela of cancer is observed in up to 80% of cancer patients and is the direct cause of mortality in up to 30% of cancer patients, contingent upon the oncological setting (Fearon et al., 2011; Fearon et al., 2012). However, until recently, very few reports have examined cachexia in the settings of ovarian cancer. Ovarian cancer is the most lethal gynecological malignancy and the fifth leading cause of cancer-related deaths amongst women in the United States (Siegel et al., 2019). A recent meta-analysis reported that cachexia is observed in 11–54% of ovarian cancer patients, although it was noted that this wide range could be attributable to different cut-off points for assessing the induction of sarcopenia in patients (Ubachs et al., 2019).

The development of a cachectic state in cancer patients is highly correlated with a decrease in quality of life, tumor resurgence, and the development of resistance to chemotherapeutic agents (Fearon et al., 2011; Fearon et al., 2012; Johns et al., 2013). Indeed, in the settings of ovarian cancer, cachexia usually accompanies the onset of chemotherapeutic resistance and the development of ascites (Barreto et al., 2016). Current first-line therapy for ovarian cancer patients (cytoreductive surgery followed by treatment with a platinum-based antineoplastic agent) initially shows a high response rate, but approximately 70% of patients will relapse and develop resistance to platinum therapy (Cannistra, 2004; Ozols et al., 2003). Both primary debulking surgery and treatment with chemotherapeutic agents (such as cisplatin) are independently associated with the induction of cachexia and are associated with a decline in ovarian cancer patient survival (Huang et al., 2020). To date, no clinically efficacious treatment is available for the treatment of cachexia.

Three recent preclinical reports have examined cachexia in the settings of ovarian cancer, one of which was from our laboratory (Pettersen et al., 2020; Pin et al., 2018; Straughn and Kakar, 2019). While different ovarian cancer cell lines were utilized in the studies, interleukin-6 (IL-6) was examined to varying degrees in all three studies, likely due to its known roles in ovarian cancer progression (Dijkgraaf et al., 2012) and its association with the induction of cachexia (Bonetto et al., 2012). Quite interestingly, the report from Pettersen et al. demonstrated that intra-tumoral Activin A signaling promotes the secretion of IL-6 from ovarian cancer cells, and that inhibiting this signaling axis can reduce the ability of cancer cells to accelerate autophagy and impede the induction of cachexia (Pettersen et al., 2020). Along similar lines, work from our laboratory demonstrated that treatment of tumor-bearing mice with Withaferin A (WFA) at one concentration results in both the reduction in various proinflammatory cytokines and an improvement in muscle strength (Straughn and Kakar, 2019). The works by Pin et al. (2018) and Straughn and Kakar (2019) suggest that preservation of muscle mass could result in improvements in patient quality of life and survival and conclude that further research into the mechanisms responsible for muscle wasting in the settings of ovarian cancer is of the utmost clinical importance.

Skeletal muscle mass is primarily regulated by (1) myogenic progenitors, (2) the rate of protein degradation, and (3) the rate of protein synthesis (Schiaffino et al., 2013). Satellite cells are the primary myogenic progenitors responsible for the majority of skeletal muscle regeneration (Kuang et al., 2007; Relaix and Zammit, 2012), and have been shown to be spuriously activated in an NF-κB-dependent manner in multiple models of cancer-induced cachexia (He et al., 2013). While an increase in proliferating satellite cells has been evidenced in the settings of cancer-induced cachexia, it was simultaneously demonstrated that they are functionally inactivated (i.e., did not differentiate/fuse with muscle to repair injury) through a Pax7-dependent downregulation of MyoD, leading to a failure in muscle repair (He et al., 2013).

Additionally, cancer-induced cachexia has been reported to upregulate various branches of the unfolded protein response (UPR), exerting deleterious effects on muscle mass (Bohnert et al., 2016; Bohnert et al., 2018). The protein kinase R-like endoplasmic reticulum kinase (PERK) arm of the UPR is required for both the survival and differentiation of satellite cells to facilitate proper muscle repair (Xiong et al., 2017). However, overactivation of the UPR is known to result in skeletal muscle atrophy through activation of proteolytic systems (Afroze and Kumar, 2019). Upregulation of both the ubiquitin proteasome system (UPS) and autophagy-lysosomal system (ALS) have been observed in skeletal muscle in the settings of cancer-induced cachexia, facilitating proteolytic degradation of proteins culminating in the atrophy of muscle (Sandri, 2010; Sandri, 2016). In addition to protein degradation modalities, the UPR also acts to limit the rate of protein synthesis through an inhibition in translation and regulation of the Akt/mTOR pathway, attenuating muscle mass (Qin et al., 2010).

In our efforts to identify a potential therapeutic agent to treat cachexia, we explored the possibility of WFA as a drug to target cachexia. WFA is a steroidal lactone that is purified from the plant *Withania somnifera*, and is not currently known to have an anabolic effect on skeletal muscle. WFA is known for its inhibitory effects on the proliferation and survival of various cancer cells (Chandrasekaran et al., 2018; Chang et al., 2017; Kyakulaga et al., 2018; Royston et al., 2018; Xia et al., 2018; Zhang and Zhang, 2017), including ovarian cancer (Fong et al., 2012; Kakar et al., 2012, 2014, 2016, 2017). WFA has been shown to induce apoptosis of both ovarian cancer cells and cancer stem cells (Kakar et al., 2017), at least in part due to a rampant increase in the production of reactive oxygen species and subsequent DNA damage (Fong et al., 2012). Interestingly, a recent report has shown in multiple breast cancer cell lines that WFA activates autophagy in the cancer cells in a UPR-dependent manner (Ghosh et al., 2017). Notably missing from this report is the effect that WFA has on UPR signaling and autophagy in non-tumorigenic cells/tissues. Recent work from our group has shown that WFA is capable of attenuating the cachectic phenotype induced by a xenograft model of ovarian cancer using the A2780 cell line (Straughn and Kakar, 2019), which is genetically similar to endometrioid-type ovarian cancer (Domcke et al., 2013; Hernandez et al., 2016).

In the present study, we sought to corroborate our prior findings that WFA treatment attenuates the atrophying and weakening effects of a xenograft model of ovarian cancer, elucidate the mechanisms by which ovarian cancer induces a cachectic phenotype, and investigate if WFA would improve functional muscle strength or size in a tumor-free setting. Further, we assessed the effect of WFA on select critical regulators of skeletal muscle, namely satellite cells and signaling to the UPS and ALS through the UPR. Promisingly, WFA treatment led to significant improvements in muscle grip strength, myofibrillar cross-sectional area (CSA), and the minimal Feret’s diameter in both tumor-free and tumor-bearing mice. Similar to published reports (He et al., 2013), our xenograft model of ovarian cancer led to a robust activation of satellite cells without improvements in myofibrillar size or muscle strength in female NSG mice, underlying a common assault to myogenic progenitors. Interestingly, WFA was found to be a more potent activator of satellite cells than the A2780 ovarian cancer xenografts. Further, we report that WFA treatment and our xenograft model differentially regulate the UPR pathways in skeletal muscle. WFA appears to produces an adaptive UPR through slight elevation in global UPR activation and robust activation of the IRE1α arm, whereas the A2780 xenografts resulted in muscle atrophy through an activation of the UPS and ALS, due to the induction of a maladaptive UPR. Summarily, WFA is a novel regulator of skeletal muscle mass that attenuates the effects of ovarian cancer-induced cachexia.

## Materials and Methods

### Cell Line

The A2780 ovarian cancer cell line was maintained in Roswell Park Memorial Institute (RPMI) Medium-1640 supplemented with: 10% Fetal Bovine Serum (FBS, Hyclone), 100 U/ml Penicillin, and 10 μg/ml Streptomycin. Cells were cultured in a humidified atmosphere of 5% CO_2_ at 37°C, and the medium was changed every 48 h as described previously (Kakar et al., 2017). The A2780 ovarian cancer cell line is of human origin, necessitating the utilization of immune-deficient mice.

### Generation of Tumor in Mice

As described previously (Kelm et al., 2020; Straughn and Kakar, 2019), six-week-old female NOD.Cg-*Prkdc^scid^ Il2rg^TM 1Wjl^*/SzJ (NSG, Jackson Lab Strain #005557) immunodeficient mice were initially randomly assigned to a tumor-free or tumor-bearing group (30 mice/group). Tumor-bearing groups received an intraperitoneal (i.p.) injection of 8.0 × 10^5^ low passage A2780 cells suspended in 100 μl sterile PBS. Control group received i.p. injection of 100 μl sterile PBS alone. After an initial refractory period of 8 days, mice in both the tumor-free and tumor-bearing groups were stratified into a group that would receive vehicle injections (10% dimethyl sulfoxide, 90% glycerol trioctanoate) or one of two concentrations of WFA (2 or 4 mg/kg) via i.p. injection (10 mice/group). Injections were performed once every 3 days until the culmination of the study. Post-euthanization, several tissues were collected, weighed, snap frozen in liquid nitrogen, and then stored at −80°C for further analysis. While alive, the mice were housed in a 12-h light–dark cycle and given water and food *ad libitum*. The Institutional Animal Care and Use Committee (IACUC, protocol # 15405) and Institutional Biosafety Committee (IBC, protocol # 18-208) of the University of Louisville approved all experimental protocols in mice in advance. None of the data in these experiments include data/samples from our initial publication discussing cachexia in the context of ovarian cancer (Straughn and Kakar, 2019).

### Grip Strength Measurements

Protocol used for the measurement of grip strength was essentially similar to as described previously (Gallot et al., 2018; Straughn and Kakar, 2019). Before assessment, mice were weighed on a commercially available digital scale. Forepaw and total grip strength of mice were measured using a digital grip strength meter (Columbus Instruments, Columbus, OH, United States) and then normalized by total body weight. Before the beginning of the test, the mice were acclimatized for five minutes. The mouse was allowed to grasp the total paw pull-bar assembly, and in a separate experiment the forepaw pull-bar assembly. The mouse was then gently drawn with constant force in a straight line away from the device until the mouse could no longer grasp the bar. Force at time of release was recorded as the peak tension. Each mouse was tested five times with a delay of 20–40 s between each testing. The mean peak tension was calculated from the recordings normalized by total body weight at time of recording. Forelimb and total limb grip strength were assessed on a weekly basis to balance elucidating functional muscle changes in response to tumor burden and WFA treatment, while simultaneously avoiding habituation to the grip strength analyses.

### Total RNA Extraction and qPCR

Isolation of total RNA from the gastrocnemius (GA) was performed using an RNeasy Fibrous Tissue Mini Kit (Qiagen Catalog # 74704) according to the manufacturer’s instructions. Skeletal muscle RNA was treated with an RNase-free DNase kit (RNase-Free DNase Set, Qiagen Catalog # 79254) in column according to the manufacturer’s instructions. First strand cDNA was synthesized using 1 μg of purified RNA and a commercially available kit (iScript^TM^ cDNA synthesis, Bio-Rad Catalog # 170-8891). Quantification of mRNA expression was performed similar to as previously described (Hindi and Kumar, 2016; Fong et al., 2012) using the SYBR Green dye method on a 7300 Real-Time PCR system (Applied Biosystems) using gene-specific primers, detailed in Supplementary Table 1.

### Histology and Morphometric Analysis of Skeletal Muscle

Select muscles of the lower limb of the mice were isolated, flash frozen in liquid nitrogen, mounted in optimal cutting temperature (OCT) embedding medium, and then sectioned using a microtome cryostat. To assess tissue morphology, 10 μm thick transverse sections were cut from the mid-belly of the tibialis anterior (TA) and then subjected to Hematoxylin and Eosin (H&E) staining. Images of H&E-stained TA muscle sections were quantified using Fiji software (National Institute of Health software) to measure myofiber CSA. Skeletal myofiber CSA was calculated by analyzing ∼500–700 myofibers per muscle as previously described (Kakar et al., 2012).

### Immunohistochemical Staining of Skeletal Muscle for the Detection of Satellite Cells and Quantification

To detect changes in the number and myogenic status of satellite cells, 8 μm thick transverse sections were cut from the mid-belly of the TA. Slides were fixed in freshly prepared 3.7% Formalin solution for 5 min. Slides were subsequently washed in 0.3 M Glycine in PBS for 10 min. Slides were then briefly washed in 0.1% Triton in PBS. Slides were then incubated for 10 min in 0.1% Triton in PBS. Slides were then subjected to an antigen retrieval step. Briefly, slides were incubated in Coplin staining jars containing 0.01 M citrate buffer and placed inside of a pressure cooker. Slides were incubated on “High Pressure” for 10 min. Slides were cooled and then washed in PBS. Slides were then blocked in a PBS solution containing 3% bovine serum albumin, 2% horse serum, and Mouse-on-Mouse blocking reagent (1 drop per 1.25 ml of blocking solution) for 60 min in a humidified chamber. Slides were then incubated overnight at 4°C in blocking solution containing primary antibodies against Pax7, MyoD, and Laminin proteins. The next day, slides were washed three times with PBS. Following, slides were incubated for 60 min in blocking solution containing appropriate secondary antibodies conjugated to fluorophores with minimally overlapping spectral emissions. Slides were subsequently washed three times using PBS for 5 min. Slides were then incubated for 5 min in DAPI resuspended in PBS (1:5,000). Slides were subsequently washed three times using PBS for 5 min. Refer to Supplementary Table 2 for a complete list of antibodies utilized. Four images from distinct areas of the muscle were captured for each animal. Imaging and quantification were performed by two separate people to allow for blinding of group identity through the use of an alphanumeric identifier. Group identities were unmasked after all data was quantified and submitted to the principal investigator, such that group-wise comparisons could be performed. For the analysis, the number of Laminin + myofibers was first counted, such that the data could eventually be normalized between images to account for differences in the size/area imaged. Next, distinct puncta in the Texas Red channel (corresponding to the secondary antibody employed for Pax7) were annotated on the image using Fiji software. These marks were then compared with the DAPI channel. When the mark for the Texas Red channel did not overlap with nuclear staining on the DAPI channel, the prospective satellite cell was ruled as a false positive and the identifying mark on the image was deleted. After deletion of false positives from the cell count feature, the number of satellite cells for the respective image was recorded. The frequency of Pax7+ cells per laminin + myofiber was then calculated for each image and then averaged. Next, the marks from the Texas Red channel were compared against the YFP channel (corresponding to the secondary antibody employed for MyoD). Two additional cell counts were initialized such that we could count cells that also exhibited YFP overlap (i.e., satellite cells that were Pax7+/MyoD+) vs. cells that did not have a YFP spectral emission (i.e., satellite cells that were Pax7+/MyoD-). The proportion of differentiating satellite cells and the proportion of satellite cells that were proliferating/quiescent was calculated for each of the four images and then averaged. This process was repeated for all 60 mice (totaling 240 RGBY images).

### Imaging

Slides were mounted using Eukitt Quick-hardening mounting medium (Sigma-Aldrich) and visualized at −0.4°C on a Nikon TiE 3000 inverted microscope (Nikon) equipped with a digital camera (DS-U2/L2-Ri1 digital microscope camera (Nikon) for light microscopy or DXM-1200C coded digital camera (Nikon) for fluorescent microscopy), and Nikon NIS Elements AR software (Nikon). Exposure times were consistent for each staining type. Image levels were equally adjusted using Adobe Photoshop CS6 software (Adobe) to remove non-specific background staining. Margins of cropped images are indicated by a dashed red border or a solid black line.

### Protein Extraction and Western Blotting

Quadriceps femoris (QF) samples were homogenized in chilled RIPA buffer (Sigma) supplemented with a Complete Mini Protease Inhibitor tablet (Roche Molecular Biochemicals, Indianapolis, IN, United States). Tissue lysates were centrifuged at 10,000 RPM and the supernatants were collected. Protein concentration for each sample was determined using the Bradford reagent method (Bio-Rad), according to manufacturer’s instructions. Protein lysates (50 μg) were separated on 10% SDS-PAGE gels at 100 V for 2 h. The proteins were transferred to nitrocellulose membranes at 100 V for 90 min. The membranes were blocked with 5% non-fat milk in Tris Buffered Saline supplemented with Tween-20 (0.05%; TBS-T) for 30–60 min. The membranes were then washed three times with TBS-T for 5 min. The membranes were then incubated with primary antibody at 4°C overnight. The membranes were washed three times with TBS-T for 5 min each followed by incubation in TBS-T containing horseradish peroxidase conjugated secondary antibody (1:3,000 dilution) for 1 h. The membranes were rinsed three times with TBS-T for 5 min each. Visualization of immunoreactive bands was enhanced using chemiluminescence reagents (Sigma). The membranes were stripped off using Restore^TM^ Western Blot Stripping Buffer (Thermo Scientific Catalog # 21059) for 15 min, blocked, and re-probed with horseradish peroxidase-conjugated β-Actin or GAPDH as a control to normalize loading variation. Refer to Supplementary Table 2 for a complete list of antibodies utilized.

### Graphical Display and Statistical Analysis

For the sake of transparency, the majority of the results were expressed as box-and-whisker plots with the box comprised of the first, second, and third quartiles, and the lower and upper whiskers corresponding to the minimum and maximum values, respectively, to display the entire range of data. Individual data points are depicted as black circles. Cropping of images is indicated by a dashed red border or a solid black line around the field of view. Statistical analysis of the data was performed using an unpaired two-tailed *t*-test with Welch’s correction for simple two group comparisons, a one-way analysis of variance (ANOVA) followed by Tukey’s Honestly Significant Difference Test (HSDT) *post hoc* analysis for comparisons between 3 or more groups with one experimental factor, or a two-way ANOVA followed by Tukey’s multiple comparison test *post hoc* analysis for comparisons between 4 or more groups containing two experimental factors to determine statistically significant differences between groups with GraphPad Prism 8.3.0 software for Mac (La Jolla, CA, United States). ANOVA summaries and the results of the multiple comparison tests are presented in Supplementary Material. Welch-corrected or Tukey-corrected *p*-value of <0.05 was considered statistically significant, unless otherwise specified.

### Ethics Statement

All procedures involving the usage of mice were carried out in strict accordance to the standards of the National Institute of Health guide for the care and use of laboratory animals. The Institutional Animal Care and Use Committee (IACUC, protocol # 15405) and Institutional Biosafety Committee (IBC, protocol # 18-208) of the University of Louisville approved all experimental protocols in mice in advance. No human data or tissue was used in this study.

## Results

### Withaferin A Improves Basal Grip Strength and Attenuates the Effects of Ovarian Cancer on Skeletal Muscle

**FIGURE 1:**
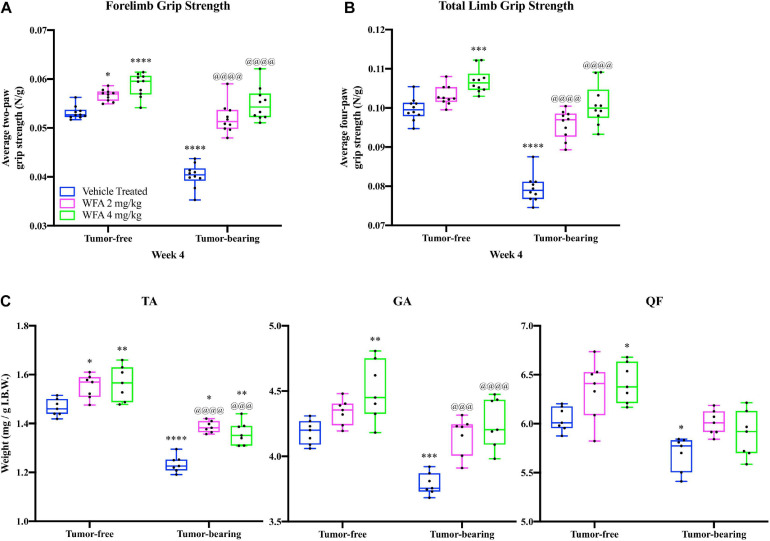
Withaferin A increases the grip strength and myofiber size of female NSG mice. Quantification of mean **(A)** forelimb and **(B)** total limb grip strength normalized to body weight in tumor-free and tumor-bearing vehicle-treated, WFA 2 mg/kg, and WFA 4 mg/kg groups at week four post-xenografting of A2780 cells. *N* = 10 in each group. **(C)** Quantification of tibialis anterior (TA), gastrocnemius (GA), and quadriceps femoris (QF) muscle wet weight normalized by initial body weight. *N* = 7 in each group. *p < 0.05; **p < 0.01; ***p < 0.001; ****p < 0.0001, value significantly different from corresponding value of tumor-free vehicle-treated group by two-way ANOVA followed by Tukey’s multiple comparison test *post hoc* analysis. ^@^*p* < 0.05, value significantly different from corresponding value of tumor-bearing vehicle-treated group.

Recent work from our lab has demonstrated that the A2780 ovarian cancer cell line is capable of inducing a skeletal muscle cachectic phenotype and that WFA attenuates these changes (Straughn and Kakar, 2019). In the present study, we attempted to elucidate whether WFA has an effect on functional muscle strength and the size of muscle in tumor-free mice, and to corroborate our prior results in tumor-bearing mice using the same lower dosage of WFA (2 mg/kg), but a different upper dosage of WFA (4 mg/kg) due to the deleterious effects previously observed with 6 mg/kg of WFA. Additionally, due to the previously reported tumor-associated mortality, the amount of xenografted cells was reduced from 1 × 10^6^ to 8 × 10^5^. A parallel report to this study from our lab examining gross body changes, tumor burden, and the effect on cardiac muscle in response to the xenografting of the A2780 ovarian cancer cell line and WFA treatment has recently been discussed in the work by Kelm et al. (2020). Information pertinent to this manuscript can be found in Figure 1, Supplementary Figure 1 and Supplementary Table 1 of the aforementioned manuscript. Before the mice received i.p. xenografts of the A2780 ovarian cancer cells, we performed forelimb and total grip strength analyses to ensure no significant differences within the groups stratified to become tumor-free or tumor-bearing existed at the start of the study (Supplementary Figures 1A,B). As expected, no significant differences were found in the baseline forelimb (tumor-free: 0.051 ± 0.004 N/g; “tumor-bearing”: 0.052 ± 0.004 N/g) or total grip strength (tumor-free: 0.094 ± 0.006 N/g; “tumor-bearing”: 0.092 ± 0.007 N/g) in the groups that were initially stratified to become tumor-free or tumor-bearing animals, as determined by an unpaired two-tailed Welch-corrected *t*-test [forelimb: *t*(57.63) = 0.74, *p* = 0.47; total limb: *t*(54.92) = 1.27, *p* = 0.21] (Supplementary Figures 1A,B). Subsequently, mice were examined for changes in grip strength once per week until the culmination of the study. Based upon the mortality rate observed in our previous study (Straughn and Kakar, 2019), the endpoint of this study was set at week 4 post-xenografting of the ovarian cancer cells. One-week post-xenografting, before WFA treatment was initiated, we observed a significant reduction in the forelimb (tumor-free: 0.056 ± 0.003 N/g; tumor-bearing: 0.049 ± 0.003 N/g) and total grip strengths (tumor-free: 0.096 ± 0.004 N/g; tumor-bearing: 0.088 ± 0.006 N/g) of the tumor-bearing mice compared to the tumor-free control group [forelimb: *t*(56.62) = 8.02; total limb: *t*(52.64) = 6.03; *p* < 0.0001 for both comparisons], suggesting a rapid onset of muscle decline (Supplementary Figures 1C,D).

At the terminal week of the study, we observed that treatment with WFA at 2 and 4 mg/kg significantly improved the forelimb grip strength of tumor-free mice (Figure 1A), and that the higher dosage of WFA had significantly improved the total grip strength of the tumor-free mice (Figure 1B) compared to the tumor-free vehicle-treated group as determined by a two-way ANOVA followed by Tukey’s multiple comparison test *post hoc* analysis (forelimb – WFA 2 mg/kg: *p* = 0.02; WFA 4 mg/kg: *p* < 0.0001; total limb – WFA 2 mg/kg: *p* = 0.30; WFA 4 mg/kg: *p* = 0.0005), suggesting that WFA treatment improves basal grip strength. Similar to our recently reported study (Straughn and Kakar, 2019), the forelimb and total limb grip strength of the tumor-bearing vehicle-treated group was significantly reduced compared to the tumor-free groups (*p* < 0.0001 for all comparisons) (Figures 1A,B). The tumor-bearing WFA-treated groups displayed forelimb and total grip strengths that were significantly increased compared to the tumor-bearing vehicle-treated (*p* < 0.0001 for all comparisons), but not significantly different than the tumor-free vehicle-treated group, corroborating our prior report that WFA ameliorates the weakening effects on skeletal muscle in our xenograft model of ovarian cancer-induced cachexia (Figures 1A,B). Grip strength analyses for week 2 and week 3 post-xenografting are presented in Supplementary Figures 1E–H. These intermediate timepoints demonstrate similar trends and p-values as the terminal week of the study.

Post-mortem, we collected select muscles of the lower extremities (TA, GA, and QF) and weighed them to detect changes in muscle mass (Figure 1C). Muscle weights were normalized by the initial body weight (IBW) to account for differences in the size of the mouse/muscle at baseline, while excluding the confounding effect of tumor burden on body weight. A reduction in the normalized weight of the TA, GA, and QF muscles was observed in the tumor-bearing vehicle-treated group compared to the tumor-free vehicle-treated group (*p* < 0.05 for all comparisons) (Figure 1C). Within the tumor-free groups, the WFA 4 mg/kg group displayed a significant increase in normalized weight of the TA, GA, and QF muscles compared to the vehicle-treated group (*p* < 0.05 for all comparisons) (Figure 1C). Within the tumor-bearing groups, treatment with both concentrations of WFA led to a statistically significant increase in normalized wet weight of the TA and GA muscles compared to the vehicle-treated group (Figure 1C). Curiously, these results are in contrast with those from our prior publication, where we did not observe significant differences in muscle mass (Straughn and Kakar, 2019). Experimental differences between the two studies, such as the initial cancer cell xenograft volume and duration of treatment could account for the seemingly discrepant results. It is also possible that the relatively small sample size in the first study did not allow us to observe small differences in muscle mass.

**FIGURE 2:**
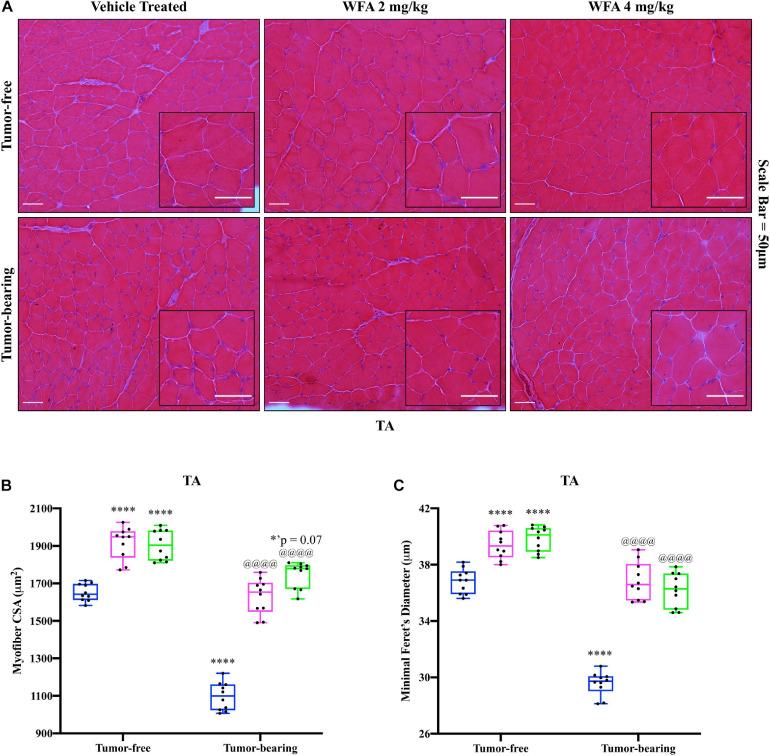
Withaferin A rescues skeletal muscle at the structural level. **(A)** Representative images of H&E-stained transverse TA muscle sections. Scale bar = 50 μm. Inset images magnified from whole image. Quantification of average **(B)** myofiber cross-sectional area (CSA) and **(C)** minimal Feret’s diameter in TA muscle. *N* = 10 in all groups. *p < 0.05; **p < 0.01; ***p < 0.001; ****p < 0.0001, value significantly different from corresponding value of tumor-free vehicle-treated group by two-way ANOVA followed by Tukey’s multiple comparison test *post hoc* analysis. ^@^*p* < 0.05, value significantly different from corresponding value of tumor-bearing vehicle-treated group.

In an attempt to corroborate the changes in muscle strength and weight in response to WFA treatment and the ovarian cancer cells, changes in myofibrillar size in the TA muscle were examined via H&E staining (Figure 2A). Similar to the changes in grip strength observed, both concentrations of WFA led to a significant increase in the average myofibrillar CSA in the TA muscle of the tumor-free WFA-treated groups (WFA 2 mg/kg: 1,916.39 ± 85.72 μm^2^; WFA 4 mg/kg: 1,904.98 ± 80.50 μm^2^) compared to the tumor-free vehicle-treated group (1,652.48 ± 46.13 μm^2^) (*p* < 0.0001 for both comparisons) (Figure 2B). The average CSA of the tumor-bearing vehicle-treated group (1,097.28 ± 74.60 μm^2^) was significantly decreased compared to all tumor-free groups (*p* < 0.0001 for all comparisons) (Figure 2B). Within the tumor-bearing groups, both dosages of WFA led to a complete rescue in myofibrillar CSA (WFA 2 mg/kg: 1,629.72 ± 94.96 μm^2^; WFA 4 mg/kg: 1,748.13 ± 68.90 μm^2^) compared to the tumor-bearing vehicle-treated group (*p* < 0.0001 for both comparisons) (Figure 2B). The average myofibrillar CSA of the tumor-bearing WFA-treated groups was not significantly different from that of the tumor-free vehicle-treated group (WFA 2 mg/kg: *p* = 0.99; WFA 4 mg/kg: *p* = 0.07) (Figure 2B). To confirm accurate measurement of the CSA, the minimal Feret’s diameter was measured in conjunction with the CSA (Figure 2C). Nearly identical trends and levels of significance in the minimal Feret’s diameter were observed compared to the CSA, indicating the validity of our histological assessment (Figure 2C). While we observed changes in myofiber size, muscle mass, and grip strength, it cannot be stated definitively that these are linked. Indeed, whether or not a correlation between muscle size and muscle functions exists is currently unresolved and under intense debate within the current literature (Loenneke et al., 2019; Taber et al., 2019).

### Withaferin A Modulates Satellite Cell Activation and Differentiation

Dysregulation of NF-κB signaling has been shown to spuriously activate satellite cells and lead to an impairment of skeletal muscle repair (He et al., 2013; Straughn et al., 2019). Previous work from our lab demonstrated that WFA treatment results in a reduction in activation/nuclear translocation of the canonical NF-κB signaling pathway component p65 within the xenografted tumor (Straughn and Kakar, 2019). However, we did not assess changes in NF-κB signaling within skeletal muscle in our prior study. Thus, we performed western blotting against phospho- and total levels of p65 in protein extracts from the QF muscle (Supplementary Figures 2A,B). We observed a significant increase in the ratio of phosphorylated p65 to total p65 protein in the tumor-bearing vehicle-treated group (Supplementary Figure 2B). This increase in NF-κB activation was ameliorated upon treatment with WFA group (Supplementary Figure 2B). Next, we assessed for changes in the IKK complex and RelA at a transcriptional level in the GA muscle (Supplementary Figure 2C). The relative transcript levels of *RelA* and *IKK*β were significantly increased in the tumor-bearing vehicle-treated group compared to the tumor-free vehicle-treated group (Supplementary Figure 2C). As expected, WFA treatment in both tumor-free and tumor-bearing animals led to a significant reduction in relative transcript levels of *RelA* and *IKK*β compared to their vehicle-treated controls (Supplementary Figure 2C). Due to WFA’s known role in inhibiting canonical NF-κB signaling (Heyninck et al., 2014; Kaileh et al., 2007), and the effect ovarian cancer has on this signaling pathway (Chen et al., 2008), we investigated if WFA or our xenograft model induced the activation of satellite cells.

**FIGURE 3:**
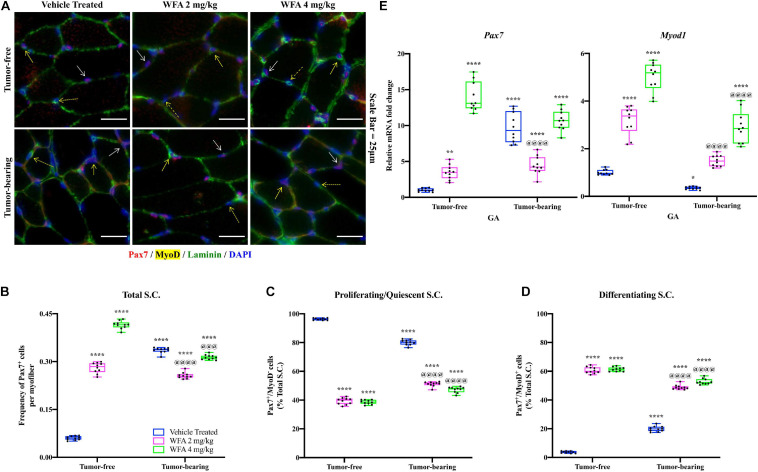
Withaferin A is a potent activator of satellite cells. **(A)** Representative images of transverse TA muscle sections after immunostaining for Pax7 (red color), MyoD (yellow color), and Laminin (green color) proteins in tumor-free and tumor-bearing groups. Nuclei were identified by counterstaining with DAPI (blue color). Scale bar = 25 μm. Quantification of **(B)** the total number of Pax7^+^ cells per Laminin^+^ myofiber, **(C)** the proportion of Pax7^+^/MyoD^–^ cells, and **(D)** the proportion of Pax7^+^/MyoD^+^ cells. *N* = 10 in all groups. Solid white arrow denotes Pax7^+^/MyoD^–^ cells. Solid yellow arrow denotes Pax7^+^/MyoD^+^ cells. Dashed yellow arrow denotes Pax7^–^/MyoD^+^ cells. **(E)** Relative mRNA levels of *Pax7* and *Myod1* in GA muscle. *N* = 10 in all groups. *p < 0.05; **p < 0.01; ***p < 0.001; ****p < 0.0001, value significantly different from corresponding value of tumor-free vehicle-treated group by two-way ANOVA followed by Tukey’s multiple comparison test *post hoc* analysis. ^@^*p* < 0.05, value significantly different from corresponding value of tumor-bearing vehicle-treated group.

The TA muscle was subjected to immunofluorescent immunohistochemical (IHC) staining for Pax7 (a marker of satellite cells), MyoD (a marker of myogenic differentiation), and Laminin (a marker of the basal lamina) (Figure 3A). As accurate IHC detection of satellite cells can be challenging, individual color channels were compared against negative control slides to optimize visualization of satellite cells, minimize non-specific background staining, and rule out false positives (Pax7^+^/DAPI^–^ cells) (Feng et al., 2018). Initially, the number of satellite cells per field (normalized by the number of Laminin^+^ myofibers) was quantified to assess whether our model of ovarian cancer or WFA treatment affected the satellite cell population at large (Figure 3B). Within the tumor-free groups, WFA treatment significantly increased the normalized amount of Pax7^+^ cells (WFA 2 mg/kg: 0.28 ± 0.02; WFA 4 mg/kg: 0.42 ± 0.01) compared to the tumor-free vehicle-treated group (0.06 ± 0.01) (*p* < 0.0001 for both comparisons) (Figure 3B). There was also a significant increase in the amount of Pax7^+^ cells in the tumor-bearing vehicle-treated group (0.34 ± 0.01) compared to the tumor-free vehicle-treated group (*p* < 0.0001) (Figure 3B). Interestingly, within the tumor-bearing groups, the WFA 2 mg/kg group displayed a significant reduction in Pax7^+^ cells (0.26 ± 0.01) compared to the vehicle-treated and WFA 4 mg/kg groups (0.31 ± 0.01) (*p* < 0.0001 for both comparisons) (Figure 3B). No definitive reason for this experimental observation has currently been elucidated. A small, but statistically significant decrease in the tumor-bearing WFA 4 mg/kg group was observed compared to the tumor-bearing vehicle-treated group (*p* = 0.0004) (Figure 2B). The tumor-free WFA 4 mg/kg group displayed the highest proportion of Pax7^+^ cells, suggesting that WFA is a potent activator of satellite cells.

After assessing changes in the gross number of satellite cells, we investigated their myogenic status to determine if the satellite cells were functionally activated. Satellite cells that are Pax7^+^/MyoD^–^ are self-renewing or returning to quiescence, whereas satellite cells that are Pax7^+^/MyoD^+^ have committed to the myogenic lineage and will facilitate in muscle repair (Kuang and Rudnicki, 2008; Kuang et al., 2007). Similar to published reports (Didier et al., 2012), the vast majority of nuclei within and outside of myofibers were found to be Pax7^–^/MyoD^+^, and as such were not considered myogenic progenitors. It was not feasible to enumerate the population of Pax7^–^/MyoD^+^ cells in response to the xenografted cancer or WFA treatment due to experimental limitations. However, distinct populations of Pax7^+^/MyoD^–^ and Pax7^+^/MyoD^+^ cells were present (Figures 3C,D). Consistent with published reports (Yoshida et al., 1998), the majority of the satellite cells in the tumor-free vehicle-treated group were Pax7^+^/MyoD^–^ (96.29 ± 0.69%) and a small population was found to be Pax7^+^/MyoD^+^ (3.71 ± 0.69%) (Figures 3C,D). The proportion of proliferating satellite cells in the tumor-bearing vehicle-treated group (80.10 ± 1.88%) was significantly decreased, and the proportion of differentiating satellite cells (19.90 ± 1.88%) was significantly increased compared to the tumor-free vehicle-treated group (Figures 3C,D). In the tumor-free groups, there was a significant decrease in the proportion of proliferating satellite cells (WFA 2 mg/kg: 39.27 ± 2.28%; WFA 4 mg/kg: 38.63 ± 1.49%) and a significant increase in differentiating satellite cells (WFA 2 mg/kg: 60.73 ± 2.28%; WFA 4 mg/kg: 61.37 ± 1.49%) in the WFA-treated groups compared to the vehicle-treated group (*p* < 0.0001 for all comparisons) (Figures 3C,D). In the tumor-bearing groups, there was a significant decrease in the proportion of proliferating satellite cells (WFA 2 mg/kg: 51.15 ± 1.63%; WFA 4 mg/kg: 47.21 ± 1.99%) and a significant increase in differentiating satellite cells (WFA 2 mg/kg: 48.85 ± 1.63%; WFA 4 mg/kg: 52.79 ± 1.99%) in the WFA-treated groups compared to the tumor-free and tumor-bearing vehicle-treated groups (*p* < 0.0001 for all comparisons) (Figures 3C,D).

To corroborate our IHC data, we performed qPCR on GA muscle extracts to assess relative transcript levels of *Pax7* and *Myod1* (Figure 3E). Similar to the IHC data, the tumor-bearing vehicle-treated group displayed a significant increase in relative transcript levels of *Pax7* and a significant decrease in *Myod1* compared to the tumor-free vehicle-treated group (Figure 3E). The tumor-free and tumor-bearing WFA-treated groups displayed significant increases in *Pax7* compared to the tumor-free vehicle-treated group and a significant increase in *Myod1* compared to both vehicle-treated groups (Figure 3E). While both WFA treatment and xenografting of ovarian cancer into mice led to an increase in satellite cell proliferation, WFA appears to signal for the differentiation of satellite cells through a mechanism not presently known.

### Withaferin A Induces an Adaptive Unfolded Protein Response

**FIGURE 4:**
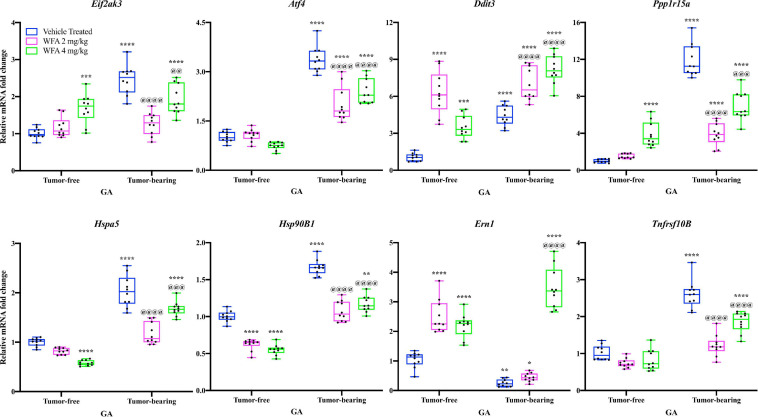
Withaferin A and ovarian cancer differentially transcriptionally regulate the UPR. Relative mRNA levels of select markers of the unfolded protein response (UPR) in the GA muscle of tumor-free and tumor-bearing groups. *N* = 10 in all groups. *p < 0.05; **p < 0.01; ***p < 0.001; ****p < 0.0001, value significantly different from corresponding value of tumor-free vehicle-treated group by two-way ANOVA followed by Tukey’s multiple comparison test *post hoc* analysis. ^@^*p* < 0.05, value significantly different from corresponding value of tumor-bearing vehicle-treated group.

It has been demonstrated in the Lewis lung carcinoma and Apc^Min/+^ models of cancer-induced cachexia that several markers of the UPR are upregulated compared to tumor-free hosts as a byproduct of increased endoplasmic reticulum (ER) stress (Bohnert et al., 2016). Due to the UPR’s role in regulating muscle mass and common perturbations associated with cancer-induced cachexia, we investigated if components of the UPR are augmented in response to WFA treatment or xenografting of the A2780 ovarian cancer cell line. Overall, both WFA and the xenografted ovarian cancer cell line led to activation of various components of the UPR in skeletal muscle, with the A2780 ovarian cancer cell line leading to a higher degree of activation than WFA treatment (Figure 4).

The relative transcript levels of *Eif2ak3* [encoding the protein PERK (Hayes et al., 1999)], *Atf4* [encoding the protein ATF4 (Karpinski et al., 1992)], *Ddit3* [encoding the protein CHOP (Marola et al., 2019)], *Ppp1r15a* [encoding the protein GADD34 (Clavarino et al., 2012)], *Hspa5* [encoding the protein GRP78 (Elfiky et al., 2020)], *Hsp90B1* [encoding the protein GRP94 (Chen et al., 2005)], and *Tnfrsf10B* [encoding death receptor 5 (DR5; Dittmann et al., 2020)] were significantly increased in the GA muscle of the tumor-bearing vehicle-treated group compared to the tumor-free vehicle-treated group (*p* < 0.0001 for all comparisons) (Figure 4). Curiously, relative transcript levels of *Ern1* [encoding inositol requiring enzyme 1α (IRE1α; Tirasophon et al., 1998)] were significantly reduced in the tumor-bearing vehicle-treated group compared to the tumor-free vehicle-treated group (*p* = 0.001) (Figure 4).

In the tumor-free mice, we observed: (1) transcriptional upregulation of *Eif2ak3*, *Ddit3*, *Ppp1r15a*, and *Ern1*, (2) downregulation of *Hspa5* and *Hsp90B1*, and (3) no change in transcript levels of *Atf4* and *Tnfrsf10B* in the WFA-treated groups compared to the vehicle-treated group (*p* < 0.001 or 0.0001 for most comparisons) (Figure 4). In the tumor-bearing mice, the WFA treatments produced a mixture of (dis)similar results. In the tumor-bearing WFA 2 mg/kg group, we observed: (1) an upregulation of relative transcript levels of *Ddit3* (*p* < 0.01), (2) a reduction in *Eif2ak3*, *Atf4*, *Ppp1r15a*, *Hspa5*, *Hsp90B1*, and *Tnfrsf10B* (*p* < 0.0001 for all comparisons), and (3) no significant difference in relative transcript levels of *Ern1* (*p* = 0.9016) compared to the tumor-bearing vehicle-treated group (Figure 4). In the tumor-bearing WFA 4 mg/kg group, we observed: (1) a significant increase in relative transcript levels of *Ddit3* and *Ern1* (*p* < 0.0001 for both comparisons) and (2) a decrease in relative transcript levels of *Eif2ak3*, *Atf4*, *Ppp1r15a*, *Hspa5*, *Hsp90B1*, and *Tnfrsf10B* (*Eif2ak3*: *p* = 0.0085; *p* < 0.001 for all other comparisons) compared to the tumor-bearing vehicle-treated group (Figure 4). The expression of *Atf6* was not detectable in a sufficient number of samples within the tumor-free or tumor-bearing groups, therefore no conclusions were drawn for this branch of the UPR (Data not shown).

**FIGURE 5:**
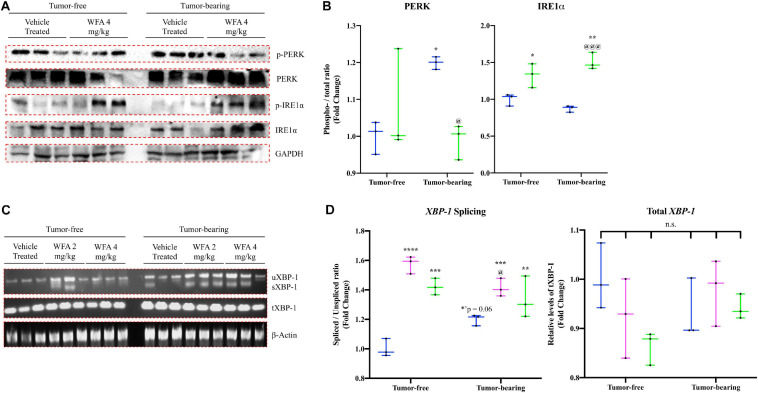
Withaferin A and ovarian cancer’s effect on the UPR at the protein level. **(A)** Representative immunoblots for components of the PERK and IRE1α arms of the UPR in QF muscle samples in the tumor-free and tumor-bearing vehicle-treated and WFA 4 mg/kg groups. *N* = 3 in all groups. **(B)** Densitometric quantification of western blot results. **(C)** Representative images of spliced (*sXBP-1*), unspliced (*uXBP-1*) and total *XBP-1* levels in GA muscle samples from tumor-free and tumor-bearing animals. Unrelated gene (β-Actin) was used as a load control for normalization. *N* = 3 per group. **(D)** Densitometric quantification of transcript levels of *XBP-1* and β-Actin. *N* = 3 per group. “n.s.” = no significant differences. *p < 0.05; **p < 0.01; ***p < 0.001; ****p < 0.0001, value significantly different from corresponding value of tumor-free vehicle-treated group by two-way ANOVA followed by Tukey’s multiple comparison test *post hoc* analysis. ^@^*p* < 0.05, value significantly different from corresponding value of tumor-bearing vehicle-treated group.

Subsequently, activation of the PERK and IRE1α arms of the UPR were assessed by Western blotting for both the phosphorylated and total protein levels of PERK and IRE1α in an attempt to corroborate the changes observed at the transcriptional level (Figures 5A,B). The ratio of phosphorylated PERK to the total levels of PERK was significantly increased in the tumor-bearing vehicle-treated group compared to the tumor-free groups, suggesting that the xenografted ovarian cancer results in activation of PERK signaling (Figure 5B). This increase in PERK activation was reversed upon treatment with WFA (4 mg/kg) and did not differ from the levels of PERK activation in the tumor-free vehicle-treated group (Figure 5B). To further assess PERK activation, western blotting against the protein CHOP was performed; however, markedly variable results were obtained in all of the groups assessed (Data not shown). The ratio of phosphorylated IRE1α to the total levels of IRE1α was significantly increased in both the tumor-free and tumor-bearing WFA 4 mg/kg groups compared to their vehicle-treated counterparts, suggesting that WFA leads to the activation of the IRE1α arm of the UPR (Figure 5B). Limitations of this experiment include the necessity to stratify macromolecular extraction to different muscles of the lower extremity due to the atrophying effects of the cancer on skeletal muscle.

In response to ER stress, IRE1α catalyzes the splicing of *XBP-1* mRNA (Bohnert et al., 2018). To further assess activation of the IRE1α arm of the UPR, we evaluated the splicing of *XBP-1* by performing semi-quantitative RT-PCR using a set of primers that detects both the unspliced (*uXBP-1*) and spliced *XBP-1* (*sXBP-1*) mRNA variants (Figure 5C). We also assessed total levels of *XBP-1* using a primer sequence that detects a conserved region shared between the spliced and unspliced variants of *XBP-1*, as well as the unrelated gene β-Actin to use as a control. Representative images for these experiments are presented in Figure 5C. The ratio of *sXBP-1* to *uXBP-1* was significantly increased in all of the WFA-treated groups, and a trend toward increase in the tumor-bearing vehicle-treated group (*p* = 0.06) was observed compared to the tumor-free vehicle-treated group (Figure 5D). After normalization by β-Actin, no significant differences in the total amount of *XBP-1* was observed between any of the groups (Figure 5D). Based upon the qPCR data and the western blotting results, it appears that WFA and our model of ovarian cancer are antagonistically acting upon the UPR to induce an adaptive or maladaptive response, respectively.

### Withaferin A Downregulates Activation of the Ubiquitin Proteasome System

**FIGURE 6:**
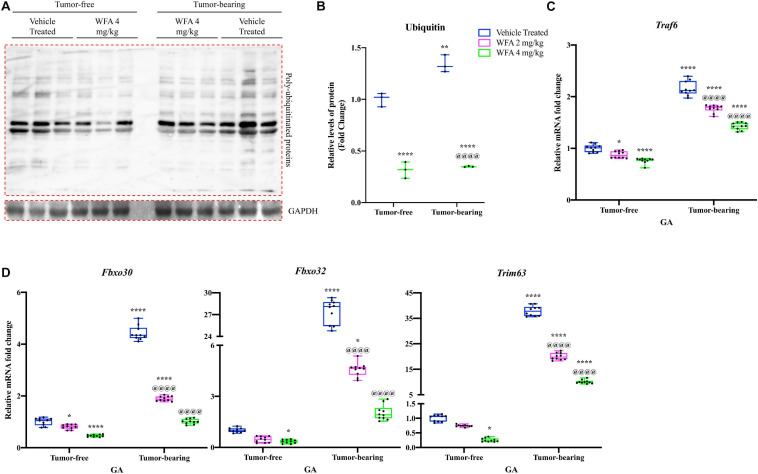
Withaferin A reduces activation of the UPS. **(A)** Representative immunoblots for poly ubiquitinated proteins and unrelated protein GAPDH in tumor-free and tumor-bearing vehicle-treated or WFA 4 mg/kg QF muscle samples. *N* = 3 per group. **(B)** Densitometric quantification of poly ubiquitinated proteins in QF muscle samples. Relative mRNA levels of **(C)** *Traf6* and **(D)** select muscle-specific E3 ubiquitin ligases in GA muscle samples. *N* = 10 in all groups. *p < 0.05; **p < 0.01; ***p < 0.001; ****p < 0.0001, value significantly different from corresponding value of tumor-free vehicle-treated group by two-way ANOVA followed by Tukey’s multiple comparison test *post hoc* analysis. ^@^*p* < 0.05, value significantly different from corresponding value of tumor-bearing vehicle-treated group.

Downstream of the UPR, the UPS is one of the two major proteolytic systems that degrades muscle proteins in oncological settings (Sandri, 2016). As an initial experiment to assess for changes in the UPS, we performed immunoblotting against ubiquitin to detect pan-levels of ubiquitination (Figure 6A). As expected, there was a significant increase in the amount of proteins in the QF muscle conjugated to ubiquitin in the tumor-bearing vehicle-treated compared to the tumor-free vehicle-treated group (Figure 6B). WFA treatment (4 mg/kg) significantly reduced the levels of ubiquitinated proteins in the tumor-free and tumor-bearing groups compared to their respective vehicle-treated control group.

We next investigated relative transcript levels of muscle-specific E3 ubiquitin ligases, as well as an E3 ubiquitin ligase that is not specific to muscle [*Traf6*, encoding the protein TRAF6 (Cao et al., 1996)] (Bodine and Baehr, 2014; Paul et al., 2012). *Fbxo30* [encoding the protein MUSA1 (Hahn et al., 2020)], *Fbxo32* [encoding the protein MAFbx (Hahn et al., 2020)], and *Trim63* [encoding the protein MuRF1 (Hahn et al., 2020)] are muscle-specific E3 ubiquitin ligases that have been shown to be involved in protein degradation under catabolic settings (Bodine and Baehr, 2014; Foletta et al., 2011). Within the tumor-free groups, there was a significant reduction in the relative transcriptional expression of *Traf6* and *Fbxo30* in the WFA 2 mg/kg group compared to the vehicle-treated group (*p* < 0.05 for both comparisons) (Figures 6C,D). The WFA 4 mg/kg group exhibited a significant reduction in relative gene expression of *Fbxo30*, *Fbxo32*, *Trim63*, and *Traf6* compared to the tumor-free vehicle-treated group (*p* < 0.0001, *p* = 0.04, *p* = 0.02, *p* < 0.0001, respectively) (Figures 6C,D). The tumor-bearing vehicle-treated group exhibited a significant upregulation in relative transcript levels of all markers of the UPS assessed compared to all tumor-free groups (*p* < 0.0001 for all comparisons) (Figures 6C,D). In the tumor-bearing animals, treatment with WFA led to a statistically significant dose-dependent transcriptional downregulation of *Fbxo30*, *Fbxo32*, *Trim63*, and *Traf6* compared to the tumor-bearing vehicle-treated group (*p* < 0.0001 for all comparisons) (Figures 6C,D). The results of these experiments suggest that modulation of the UPS could be one of the signaling pathways responsible for WFA’s positive effect on skeletal muscle mass.

### Withaferin A Transcriptionally Downregulates the Autophagy-Lysosomal System

In addition to the UPS, cancer-induced cachexia has been shown to activate the ALS to facilitate an atrophying effect on skeletal muscle (Sandri, 2010). During autophagy, LC3B-I is converted to LC3B-II to allow interaction with autophagic vesicles (Tanida et al., 2004). p62 is a substrate of autophagy that recognizes and binds to ubiquitinated proteins (Lamark et al., 2017). As such, p62 protein levels decrease with the activation of autophagy and it becomes transcriptionally upregulated to replenish the depleting protein (Lamark et al., 2017). Beclin1 is another autophagy-related protein that is critical for the initiation of the autophagosome (Sun et al., 2009).

**FIGURE 7:**
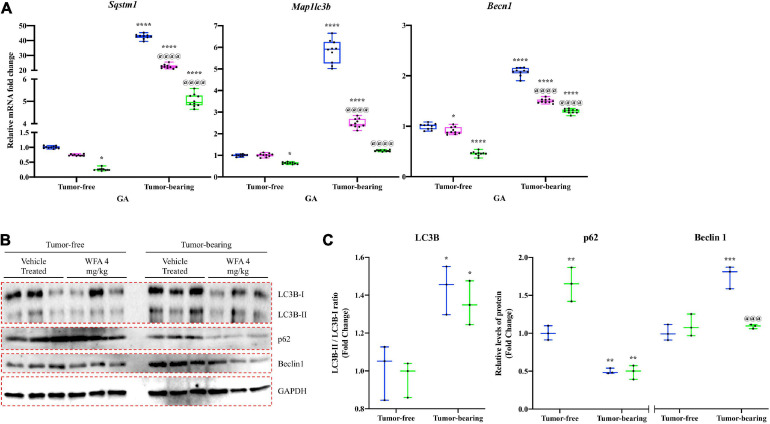
Withaferin A downregulates activation of the ALS. **(A)** Relative mRNA levels of select markers of autophagy in GA muscle samples from tumor-free and tumor-bearing mice. *N* = 10 in all groups. **(B)** Western blot analysis of common markers of autophagy in QF muscle samples. *N* = 3 per group. **(C)** Densitometric quantification of western blot images. *p < 0.05; **p < 0.01; ***p < 0.001; ****p < 0.0001, value significantly different from corresponding value of tumor-free vehicle-treated group by two-way ANOVA followed by Tukey’s multiple comparison test *post hoc* analysis. ^@^*p* < 0.05, value significantly different from corresponding value of tumor-bearing vehicle-treated group.

To examine the effect of WFA and our xenograft model on the ALS, we measured relative mRNA levels of common autophagy markers: *Sqstm1* [encoding the protein p62 (Sánchez-Martín and Komatsu, 2018)], *Map1lc3b* [encoding the protein LC3B (Kang et al., 2019)], and *Becn1* [encoding the protein Beclin1 (Zheng et al., 2020)] (Figure 7A). A significant reduction in relative gene expression of the aforementioned markers of the ALS was observed in the tumor-free WFA 4 mg/kg group compared to the tumor-free vehicle-treated group (*p* = 0.04, *p* = 0.013, *p* < 0.0001, respectively) (Figure 7A). In the tumor-bearing vehicle-treated group, we observed a significant increase in relative transcript levels of *Sqstm1*, *Map1lc3b*, and *Becn1* compared to all tumor-free groups (*p* < 0.0001 for all comparisons) (Figure 7A). Similar to the results for the UPS, WFA treatment led to a significant dose-dependent reduction in relative mRNA expression of the select ALS markers compared to the tumor-bearing vehicle-treated group (*p* < 0.0001 for all comparisons) (Figure 7A).

Subsequently, we performed immunoblotting against LC3B, p62, and Beclin1 to determine whether WFA or ovarian cancer augmented signaling to the ALS at a protein level (Figures 7B,C). The ratio of LC3B-II to LC3B-I was significantly increased in the tumor-bearing groups compared to the tumor-free groups. However, WFA treatment did not affect LC3B lipidation under tumor-free or tumor-bearing conditions. Interestingly, protein levels of p62 were significantly increased in the tumor-free WFA 4 mg/kg compared to the tumor-free vehicle-treated group, suggesting a reduction in basal levels of autophagy (Figure 7C). A significant reduction in the levels of p62 in the tumor-bearing vehicle-treated and WFA 4 mg/kg groups was observed compared to the tumor-free vehicle-treated group, suggesting an active autophagy process was occurring (Figure 7C). Further, we observed a significant increase in Beclin1 only in the tumor-bearing vehicle-treated group compared to the tumor-free vehicle-treated group (Figure 7C). The tumor-bearing WFA 4 mg/kg group had a significant reduction in Beclin1 protein levels compared to the tumor-bearing vehicle-treated group (Figure 7C). Similar to the UPS, the results from these experiments suggest that ovarian cancer results in a hyper-catabolic state. However, WFA appears to only regulate the ALS at a transcriptional level.

## Discussion

Despite the breadth of preclinical studies investigating cancer-induced cachexia, no pharmacological intervention has been found to be efficacious, with respect to skeletal muscle preservation/recovery, when employed in clinical trials (Naito, 2019). Several pharmacological interventions or targets for therapy are under investigation, with megestrol acetate perhaps being the most well-known (Gullett et al., 2011; Zhang et al., 2018). While megestrol acetate is used clinically to improve appetite and increase total body weight in cancer cachexia patients, its effect on skeletal muscle mass is minimal at best (Gullett et al., 2011). In order to improve the survival rates of cancer patients and preserve quality of life, it is critical that muscle mass be preserved. Prospective interventions could achieve this through regulation of tumor burden or by acting on skeletal muscle itself.

It was previously thought that tumor burden correlated with the induction and severity of cachexia, and that reducing gross tumor burden could rescue muscle mass (De Lerma Barbaro, 2015). Some studies continue to support this hypothesis (Wang et al., 2011; Wyke et al., 2004; Yang et al., 2013), but there are also reports that demonstrate the induction or severity of cachexia is independent of tumor burden (Goncalves et al., 2018; Williams et al., 2012). However, with respect to ovarian cancer, there is scant information available regarding the relationship between tumor burden and the induction of cachexia. Ovarian cancer is one of the most lethal gynecological malignancies and is primarily treated with an initial debulking surgery, followed by an adjuvant platinum-based chemotherapy agent, such as cisplatin (Cannistra, 2004; Ozols et al., 2003; Siegel et al., 2019). The deleterious effects of cisplatin on skeletal muscle are well documented (Conte et al., 2020; Damrauer et al., 2018; Sakai et al., 2014), and a recent report from Huang et al. has demonstrated that surgical debulking of advanced-stage ovarian cancer can lead to a reduction in skeletal muscle mass (Huang et al., 2020).

Recent work from our lab and the present study examined cachexia in response to the xenografting of ovarian cancer in a preclinical mouse model and employed WFA as an experimental therapeutic agent to attenuate cachexia. However, it was not determined in our initial study whether WFA’s positive effect on skeletal muscle occurred through a reduction in tumor burden or if WFA acted directly upon skeletal muscle (Straughn and Kakar, 2019). The results from this study clearly demonstrate that WFA treatment is capable of increasing functional muscle strength and muscle mass in both tumor-bearing and tumor-free mice, suggesting that the beneficial effect of WFA is not purely as a byproduct of its anti-tumorigenic properties (Figures 1, 2). Yet, this begs the question: how does WFA induce a hypertrophying or anti-cachectic effect on skeletal muscle? Generally, muscle mass is regulated by the pool of myogenic progenitors and the rates of protein degradation and synthesis (Schiaffino et al., 2013).

Satellite cells are the myogenic progenitor type primarily responsible for regenerative myogenesis (He et al., 2013; Relaix and Zammit, 2012). Further, multiple reports have demonstrated that tumor-bearing humans and animals exhibit destructive alterations to the sarcolemma of skeletal muscle fibers (Acharyya et al., 2005; He et al., 2013; Talbert et al., 2014). Indeed, disruption of the sarcolemma is known to activate satellite cells in an effort to repair damage to skeletal muscle (Charge, 2004). Disruption of the sarcolemma within skeletal muscle is thought to occur in response to tumor factors in circulation, as opposed to direct infiltration of the muscle by metastatic cancer cells (He et al., 2013). As such, satellite cells were high on our list of possible targets being augmented by WFA and/or the xenografted ovarian cancer cells. Both inoculation with A2780 ovarian cancer cells and treatment with WFA led to a robust increase in the total amount of satellite cells (Figure 3). While the exact signaling mechanism responsible for the dysregulation of muscle repair is not presently known, several prevailing theories exist.

Perhaps the most well-known, dysregulation of NF-κB signaling has been shown to impair regenerative myogenesis in the settings of cancer-induced cachexia (He et al., 2013). It was demonstrated that cancer leads to an overactivation of NF-κB signaling, leading to a Pax7-dependent down-regulation of MyoD (He et al., 2013). In our experiment, when satellite cells were stratified based upon their expression of MyoD, we observed markedly dichotomous results. WFA treatment in tumor-bearing and naïve mice led to a robust increase in the proportion of differentiating satellite cells (i.e., Pax7^+^/MyoD^+^), whereas the vehicle-treated groups had an overwhelming majority of satellite cells that were quiescent or returning to quiescence (i.e., Pax7^+^/MyoD^–^). Downstream of NF-κB signaling, proinflammatory cytokines (such as TNFα) and Angiotensin II (Ang-II) were found to prevent the expression of MyoD and myogenin, and thus impairs regenerative myogenesis (Szalay et al., 1997; Langen et al., 2001; Johnston et al., 2010; Yoshida et al., 2013). Indeed, reports from our lab demonstrated that xenografting of the ovarian cancer cell line A2780 results in a similar increase in several NF-κB-dependent proinflammatory cytokines and circulating levels of Ang-II (Kelm et al., 2020; Straughn and Kakar, 2019), perhaps indicating the mechanism through which satellite cells are dysregulated in response to ovarian cancer.

Our data suggests that WFA treatment leads to the functional activation of satellite cells and subsequent fusion of myogenic progenitors through an increase in the proportion of differentiating satellite cells and concomitant increase in myofibrillar CSA. However, the reported results (Figures 1, 2) are indirect measures that fusion is occurring. Future work from our lab will specifically address whether or not myogenic fusion is occurring in response to WFA treatment to discern if myogenic progenitors are indeed responsible for the beneficial effect on skeletal muscle. Aside from myogenic progenitors, changes in signaling to the UPR have been exhibited in several models of cancer-induced cachexia (Bohnert et al., 2016; Isaac et al., 2016; Roy and Kumar, 2019).

The UPR acts to resolve ER stress and to facilitate the proper folding of proteins by limiting the rate of protein synthesis and upregulating the production of chaperone proteins (Tirasophon et al., 1998; Wang and Kaufman, 2014). In circumstances where ER stress is insurmountable, the UPR will signal proteolytic pathways culminating in cellular death (Hetz, 2012). In our study, we observed that both WFA and ovarian cancer globally regulate various components of the UPR, albeit with a few critical distinctions (Figures 4, 5). WFA treatment led to a transcriptional upregulation of the IRE1α arm of the UPR, and a reduction in Death Receptor 5 expression. Further, WFA treatment resulted in a significant increase in the protein levels of phosphorylated IRE1α. Conversely, xenografting of ovarian cancer cells resulted in an increase in the PERK arm of the UPR and a concomitant increase in Death Receptor 5 expression. A significant increase in the protein levels of phosphorylated PERK was shown in response to the xenografted ovarian cancer cells, but not WFA treatment. However, discrepant results were observed when interrogating the UPR distal to PERK and IRE1α activation.

The protein CHOP is normally associated with activation of autophagy mediated through the PERK arm of the UPR, although both the IRE1α and ATF6 arms of the UPR are capable of augmenting CHOP expression (Hu et al., 2018; Nishitoh, 2012). Conflicting reports exist regarding the effect of CHOP signaling in skeletal muscle. One study found that depletion of CHOP accentuates skeletal muscle atrophy (Yu et al., 2011), whereas a different study reported no significant changes in CHOP in a model of disuse muscle atrophy (Hunter et al., 2001). Thus, it would seem that the effects of CHOP signaling appear to be context-specific. In our study, no consistent changes in the protein levels of CHOP were observed in response to WFA treatment or xenografting of the A2780 ovarian cancer cell line, although we did observe a significant increase in transcript levels of the gene encoding CHOP in response to both WFA treatment and the ovarian cancer cell xenografts. Activation of IRE1α arm of the UPR results in the alternative splicing of *XBP-1* transcripts. WFA treatment under tumor-free and tumor-bearing conditions resulted in a significant increase in activation of IRE1α, as well as in a significant increase in the ratio of *sXBP-1* to *uXBP-1*. Interestingly, xenografting of the ovarian cancer cells into NSG mice did not lead to a significant increase in IRE1α activation or splicing of *XBP-1*. These results suggest that, although WFA and ovarian cancer both generally activate the UPR, a differential pattern of UPR activation is exhibited in response to WFA treatment and the xenografted cancer cells within skeletal muscle.

While we corroborated immediately distal changes in signaling through the IRE1α arm of the UPR, but not the PERK arm, it was unknown whether these changes were of any consequence with respect to muscle atrophy. Thus, we examined the ubiquitin proteasome and ALSs to determine if ovarian cancer produced a hyper-catabolic state. At the protein level, both pan-ubiquitination of proteins and activation of autophagy were exhibited in skeletal muscle in response to ovarian cancer (Figures 6, 7). Transcript levels of select E3 ubiquitin ligases and markers of the ALS were similarly increased in response to the ovarian cancer cell xenografts. Interestingly, WFA treatment produced a robust transcriptional downregulation of several components of the UPS and ALS. However, WFA treatment led to a significant reduction in polyubiquitinated proteins, but only downregulated select ALS proteins. Thus, it is possible that WFA’s effect on augmenting protein degradation primarily occurs through changes in the UPS. Skeletal muscle atrophy is known to have various sex-specific effects (Rosa-Caldwell and Greene, 2019; Zhong and Zimmers, 2020). One notable example is that skeletal muscle in males tends to exhibit greater activation of the UPS, whereas skeletal muscle in females preferentially activates the ALS (Ogawa et al., 2017; Oliván et al., 2014; Piekarski et al., 2014; Shu et al., 2018). As a result of this, WFA treatment may show an even greater effect in rescuing skeletal muscle in males.

**FIGURE 8:**
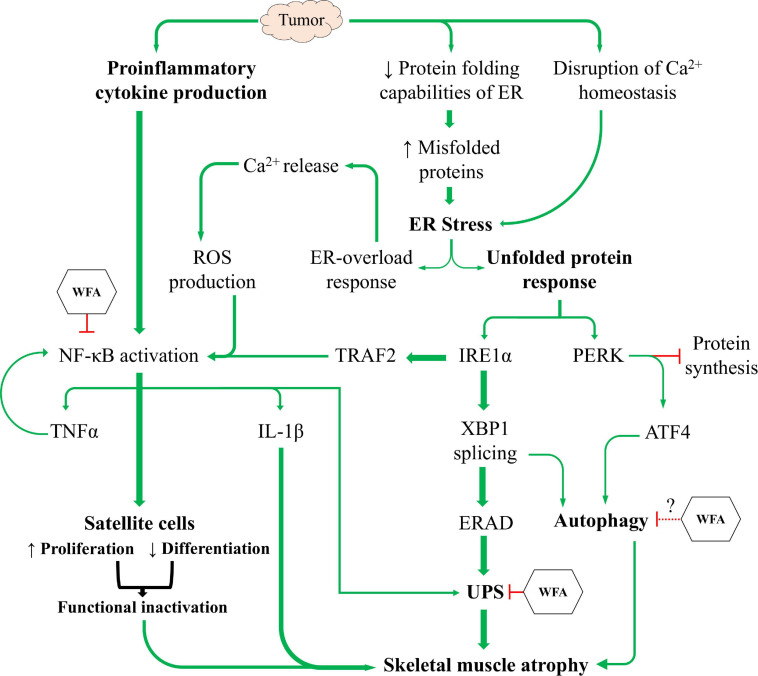
The induction of cachexia by ovarian cancer. Proposed mechanistic model showing ovarian cancer’s effect on satellite cell homeostasis and the UPR to facilitate skeletal muscle atrophy, and the proposed mechanism(s) through which WFA interferes with the induction of cachexia.

In this study, we sought to corroborate prior reports from our lab regarding the induction of cachexia by ovarian cancer and investigate signaling pathways distal to those previously reported (Kelm et al., 2020; Straughn and Kakar, 2019). Similar to other models of cancer-induced cachexia, we report that ovarian cancer induces an atrophying effect on skeletal muscle by inhibiting myogenic progenitors and activating proteolytic degradation pathways (Figure 8). Based upon current literature and our experimental results, we believe that WFA is targeting the NF-κB signaling axis, the UPS, and the ALS in an attempt to mitigate the effects of ovarian cancer in skeletal muscle. Cumulatively, our results demonstrate for the first time that WFA not only has anti-tumorigenic properties, but also directly targets skeletal muscle resulting in a hypertrophying/anti-cachectic effect. While our study omitted the inclusion of male subjects due to the anatomic origin of the cancer, it would be interesting to observe whether WFA treatment has similar effects on male subjects and whether the anti-tumorigenic and/or anti-cachectic properties carried over into a more traditional model of cachexia.

## Limitations of the Study

For studies examining changes in skeletal muscle, it is generally preferable to utilize mice that are sexually mature to exclude the potentially confounding effect of stunted growth. However, most xenograft studies employing ovarian cancer use mice between the ages of 4–8 weeks, as older mice have higher rates of xenograft rejection (Helland et al., 2014; Guo et al., 2017; De Haven Brandon et al., 2020; Shaw et al., 2004). Despite this, at least one group of investigators has compared the effects of cancer-induced cachexia in young mice vs. old mice, albeit in a different model of cancer cachexia (Talbert et al., 2014). The authors report that both young and old mice exhibit similar responses to the tumor, with respect to changes in skeletal muscle, and concluded that, “…there does not seem to be an overwhelming reason to adapt tumor models to older animals…” (Talbert et al., 2014). Second, due to the atrophying effects of the cancer upon skeletal muscle, macromolecular extractions for RNA and protein were stratified to different muscles. As such, it is currently unknown if muscle-specific effects of ovarian cancer and WFA exist, which could augment the results gathered. Third, although a tumor-free control was employed in the study to aide in examining the skeletal muscle effect of WFA, the global nature of the treatment makes it impossible to differentiate between WFA’s effect on muscle, the tumor, and global levels of inflammation within the tumor-bearing groups.

Last, a separate limitation of the study involves the mouse strain employed. Most cachexia studies utilize immune-competent mice and a mouse-derived cancer. In our study, we utilized immune-deficient mice, such that we could xenograft ovarian cancer cells of human origin. However, the immune system is well-known to be involved in muscle damage and repair (Tidball and Villalta, 2010). While the immune system is certainly involved in the progression of cachexia, it is primarily centered around the induction of systemic inflammation (Argilés et al., 2009) and tumor surveillance (Miller et al., 2019). Ovarian cancer is considered to be an immunologically “cold” tumor, as they are scarcely immunogenic and have a low number of tumor-infiltrating lymphocytes (Galon and Bruni, 2019; Ghisoni et al., 2019). Due to a loss-of-function mutation of the *Prkdc* gene, NSG mice are deficient in T-cells (Shultz et al., 1995) and can thus provide a suitable environment to produce “cold” tumors upon xenografting of ovarian cancer cells. The pro-inflammatory cytokines TNFα, IL-1β, IL-6, and IFNγ are considered to be some of the critical mediators orchestrating a cachectic phenotype (Argilés et al., 2009; Fearon et al., 2012). Despite NSG mice lacking a competent immune system, the ovarian cancer cells in our study produces all four of these critical pro-inflammatory cytokines (Straughn and Kakar, 2019; Watanabe et al., 2012). For these reasons, we believe that the phenotype exhibited in our study is fairly faithful to the cachectic phenotype exhibited in humans. However, it is equally plausible that, had immune-competent animals and a suitable mouse-derived ovarian cancer cell line been employed, the cachectic phenotype exhibited could be altered.

## Data Availability Statement

The original contributions presented in the study are included in the article/Supplementary Material, further inquiries can be directed to the corresponding author/s.

## Ethics Statement

The animal study was reviewed and approved by the University of Louisville’s Institutional Animal Care and Use Committee (IACUC).

## Author Contributions

AS conceived and designed the work, wrote the manuscript, and performed statistical analyses. AS and NK performed the experiments and data acquisition and quantified the data. NK and SK reviewed the statistical analyses. All authors edited the manuscript and read and approved the final manuscript.

## Conflict of Interest

The authors declare that the research was conducted in the absence of any commercial or financial relationships that could be construed as a potential conflict of interest. The handling editor declared a past collaboration with one of the authors AS.

## Supplementary Material

Supplementary Figure 1 Basal and intermediate grip strength analyses. Quantification of mean basal **(A)** forelimb and **(B)** total limb grip strength normalized to body weight before xenografting of A2780 cells. Quantification of mean **(C)** forelimb and **(D)** total limb grip strength normalized to body weight at week one post-xenografting of A2780 cells. *N* = 30 in all groups. Quantification of mean **(E)** forelimb and **(F)** total limb grip strength normalized to body weight at week two post-xenografting of A2780 cells. Quantification of mean **(G)** forelimb and **(H)** total limb grip strength normalized to body weight at week three post-xenografting of A2780 cells. *N* = 10 in all groups. ^∗^*p* < 0.05; ^∗∗^*p* < 0.01; ^∗∗∗^*p* < 0.001; ^****^*p* < 0.0001, value significantly different from corresponding value of tumor-free vehicle-treated group by two-way ANOVA followed by Tukey’s multiple comparison test *post hoc* analysis. ^@^*p* < 0.05, value significantly different from corresponding value of tumor-bearing vehicle-treated group.

Supplementary Figure 2 Withaferin A inhibits activation of canonical NF-κB signaling in skeletal muscle. **(A)** Representative immunoblots for phospho- and total p65, as well as unrelated protein GAPDH in QF muscle samples in the tumor-free and tumor-bearing vehicle-treated and WFA 4 mg/kg groups. *N* = 3 in all groups. **(B)** Densitometric quantification of western blotting results. **(C)** Relative mRNA levels of RelA and IKKβ in GA muscle samples from all tumor-free and tumor-bearing groups. *N* = 10 in all groups. ^∗^*p* < 0.05; ^∗∗^*p* < 0.01; ^∗∗∗^*p* < 0.001; ^****^*p* < 0.0001, value significantly different from corresponding value of tumor-free vehicle-treated group by two-way ANOVA followed by Tukey’s multiple comparison test *post hoc* analysis. ^@^*p* < 0.05, value significantly different from corresponding value of tumor-bearing vehicle-treated group.
