# Supplementary figures and images for: Withaferin A and Ovarian Cancer Antagonistically Regulate Skeletal Muscle Mass
Source: Front Cell Dev Biol. 2021 Feb 25;9:636498. doi: 10.3389/fcell.2021.636498 (PMC7947350; doi:10.3389/fcell.2021.636498)

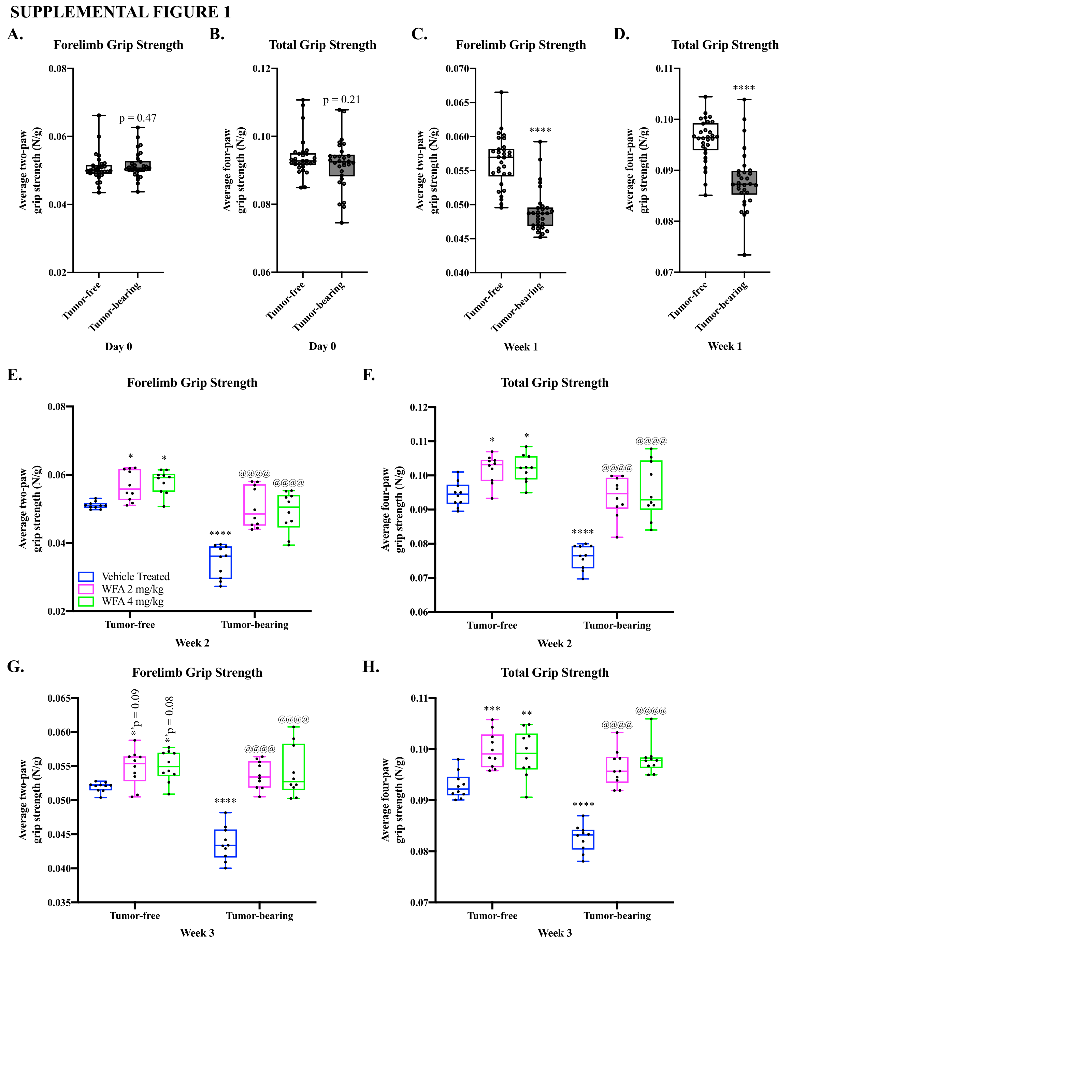

Supplement: Supplementary Figure 1 — Basal and intermediate grip strength analyses. Quantification of mean basal (A) forelimb and (B) total limb grip strength normalized to body weight before xenografting of A2780 cells. Quantification of mean (C) forelimb and (D) total limb grip strength normalized to body weight at week one post-xenografting of A2780 cells. N = 30 in all groups. Quantification of mean (E) forelimb and (F) total limb grip strength normalized to body weight at week two post-xenografting of A2780 cells. Quantification of mean (G) forelimb and (H) total limb grip strength normalized to body weight at week three post-xenografting of A2780 cells. N = 10 in all groups. ∗p < 0.05; ∗∗p < 0.01; ∗∗∗p < 0.001; ****p < 0.0001, value significantly different from corresponding value of tumor-free vehicle-treated group by two-way ANOVA followed by Tukey’s multiple comparison test post hoc analysis. @p < 0.05, value significantly different from corresponding value of tumor-bearing vehicle-treated group. [file Image_1.TIFF]

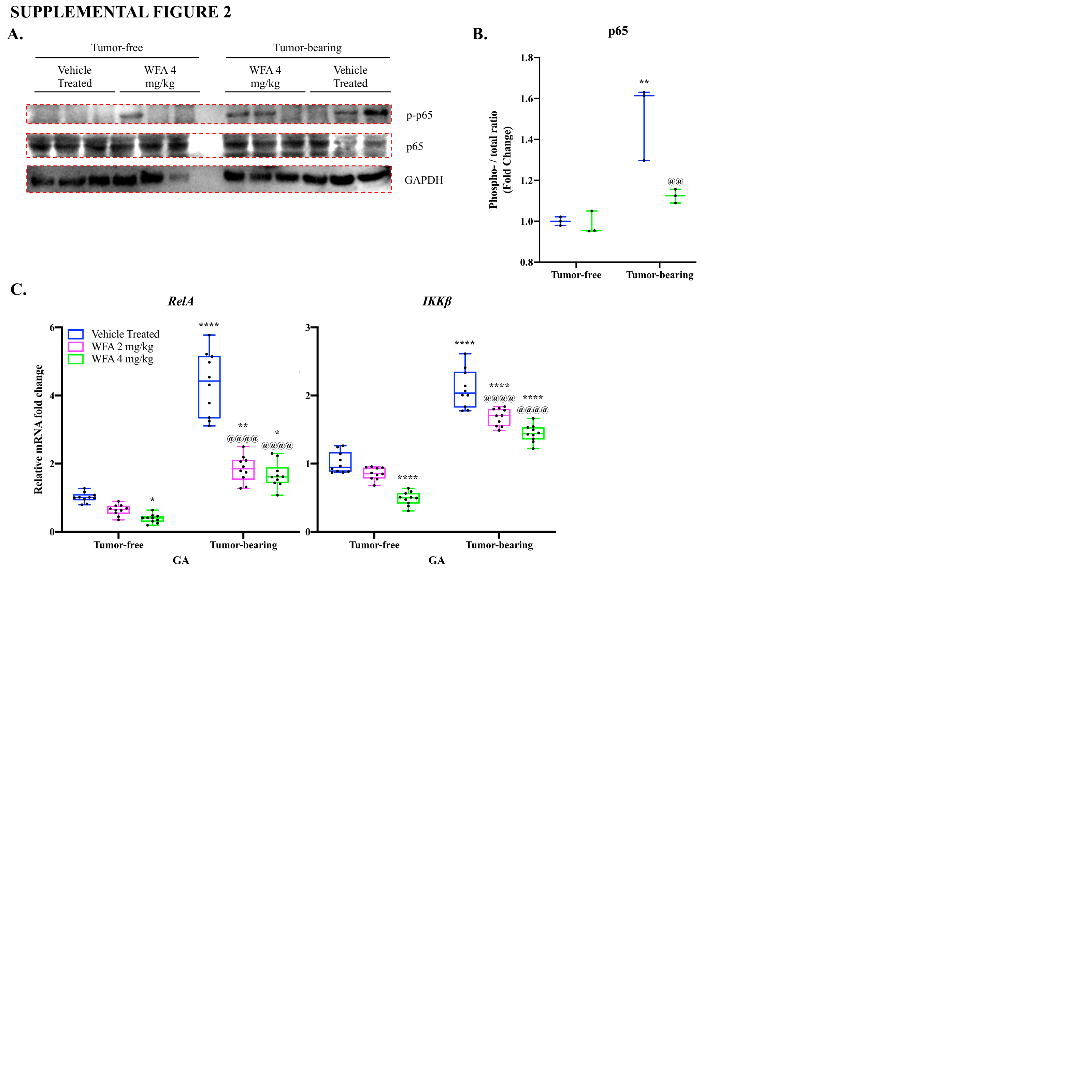

Supplement: Supplementary Figure 2 — Withaferin A inhibits activation of canonical NF-κB signaling in skeletal muscle. (A) Representative immunoblots for phospho- and total p65, as well as unrelated protein GAPDH in QF muscle samples in the tumor-free and tumor-bearing vehicle-treated and WFA 4 mg/kg groups. N = 3 in all groups. (B) Densitometric quantification of western blotting results. (C) Relative mRNA levels of RelA and IKKβ in GA muscle samples from all tumor-free and tumor-bearing groups. N = 10 in all groups. ∗p < 0.05; ∗∗p < 0.01; ∗∗∗p < 0.001; ****p < 0.0001, value significantly different from corresponding value of tumor-free vehicle-treated group by two-way ANOVA followed by Tukey’s multiple comparison test post hoc analysis. @p < 0.05, value significantly different from corresponding value of tumor-bearing vehicle-treated group. [file Image_2.TIFF]
