# Supplementary material for: Withaferin A and Ovarian Cancer Antagonistically Regulate Skeletal Muscle Mass
Source: Front Cell Dev Biol. 2021 Feb 25;9:636498. doi: 10.3389/fcell.2021.636498 (PMC7947350; doi:10.3389/fcell.2021.636498)
Supplement: Supplementary file 5 [file Data_Sheet_3.docx]

**Supplementary Table 1**: Gene specific primer sequences.

| **Gene** | **Species** | **Forward** | **Reverse** |
| --- | --- | --- | --- |
| *Pax7* | Mus musculus | 5’-CAGTGTGCCATCTACCCATGCTTA-3’ | 5’-GGTGCTTGGTTCAAATTGAGCC-3’ |
| *Myod1* | Mus musculus | 5’-TGGGATATGGAGCTTCTATCGC-3’ | 5’-GGTGAGTCGAAACACGGATCAT-3’ |
| *Eif2ak3* | Mus musculus | 5’-ACTCCTGTCTTGGTTGGGTCTGAT-3’ | 5’-CGTGCTCCGCTTATTCCTTTCT-3’ |
| *Atf4* | Mus musculus | 5’-CTCTTCACGAAATCCAGCAGCA-3’ | 5’-CCATGAGGTTTCAAGTGCTTGG-3’ |
| *Ddit3* | Mus musculus | 5’-TGAAAGCAGAACCTGGTCCA-3’ | 5’-CACTGTTCATGCTTGGTGCA-3’ |
| *Ppp1r15a* | Mus musculus | 5’-CAGAACATCAAGCCACGGAAGA-3’ | 5’-AAAGTTGTCTCAGGTCCTCCTTCC-3’ |
| *Hspa5* | Mus musculus | 5’-CGTGGAGATCATAGCCAACGAT-3’ | 5’-ATTCCAAGTGCGTCCGATGA-3’ |
| *Hsp90B1* | Mus musculus | 5’-GGGAGGTCACCTTCAAGTCG-3’ | 5’-CTCGAGGTGCAGATGTGGG-3’ |
| *Ern1* | Mus musculus | 5’-AAAGGTTCCGCTCATACAAAGGG-3’ | 5’-TGAATGAAGCCAGCAGGAAGTTG-3’ |
| *Tnfrsf10B* | Mus musculus | 5’-AAGCCTTGCAGAGAGGGTATTGAC-3’ | 5’-GCAGTTAGAGCATGACTG GCAGAT-3’ |
| *Fbxo30* | Mus musculus | 5’-TCGTGGAATGGTAATCTTGC-3’ | 5’-CCTCCCGTTTCTCTATCACG-3’ |
| *Fbxo32* | Mus musculus | 5’-AAGGCTGTTGGAGCTGATAGCA-3’ | 5’-CACCCACATGTTAATGTTGCCC-3’ |
| *Trim63* | Mus musculus | 5’-TAACTGCATCTCCATGCTGGTG-3’ | 5’-TGGCGTAGAGGGTGTCAAACTT-3’ |
| *Traf6* | Mus musculus | 5’-TCGCCTAGTAAGACAGGACCATCA-3’ | 5’-TGGTTCCATTTCGGCAACCT-3’ |
| *Sqstm1* | Mus musculus | 5’-AGCACAGGCACAGAAGACAAGAGT-3’ | 5’-AATGTGTCCAGTCATCGTCTCCTC-3’ |
| *Map1lc3b* | Mus musculus | 5’-CTGGTGAATGGGCACAGCATG-3’ | 5’-CGTCCGCTGGTAACATCCCTT-3’ |
| *Becn1* | Mus musculus | 5’-TGAAATCAATGCTGCCTGGG-3’ | 5’-CCAGAACAGTATAACGGCAACTCC-3’ |
| *RelA* | Mus musculus | 5’-TGACCCCTGTCCTCTCACATCCG-3’ | 5’-CAGCTCCCAGAGTTCCGGTT-3’ |
| *IKKβ* | Mus musculus | 5’-CTGAAGATCGCCTGTAGCAAA-3’ | 5’-TCCATCTGTAACCAGCTCCAG-3’ |
| β-Actin | Mus musculus | 5’-CAGGCATTGCTGACAGGATG-3’ | 5’-TGCTGATCCACATCTGCTGG-3’ |
| *XBP1*  (u/s) RT | Mus musculus | 5’-TTACGGGAGAAAACTCAGGGC-3’ | 5’-GGGTCCAACTTGTCCAGAATGC-3’ |
| *XBP1*  (total) RT | Mus musculus | 5’-TGGAGCAGCAAGTGGTGGATTT-3’ | 5’-TGTCCATTCCCAAGCGTGTTCT-3’ |

**Supplementary Table 2:** Primary and secondary antibody list.

| **Antibody** | **Company** | **Catalog #** | **Application** | **Dilution** |
| --- | --- | --- | --- | --- |
| Pax7 (IgG1) | Santa Cruz | sc-81648 | IHC | 1:10 |
| MyoD (IgG2b) | Santa Cruz | sc-377460 | IHC | 1:20 |
| Laminin (Rabbit) | Sigma Aldrich | L9393 | IHC | 1:100 |
| Anti-mouse IgG1 CF594 | Sigma Aldrich | SAB4600326 | IHC | 1:2000 |
| Goat anti-rabbit Alexa Fluor 488 | Thermo Fisher Scientific | A-11008 | IHC | 1:2000 |
| Goat anti-mouse IgG2b Alexa Fluor 546 | Thermo Fischer Scientific | A-21143 | IHC | 1:2000 |
| DAPI | Roche | 10236276001 | IHC | 1:5000 |
| Phospho-PERK (p-Thr980) | Invitrogen | MA5-15033 | WB | 1:500 |
| PERK | Santa Cruz | sc-377400 | WB | 1:1000 |
| Phospho-IRE1α (p-Ser724) | GeneTex | GTX132808 | WB | 1:500 |
| IRE1α (HRP Linked) | Santa Cruz | sc-390960 HRP | WB | 1:1000 |
| Ubiquitin | Cell Signaling Technologies | 3933 | WB | 1:2000 |
| LC3B | Cell Signaling Technologies | 3868 | WB | 1:1000 |
| Beclin 1 (HRP Linked) | Santa Cruz | sc-48341 HRP | WB | 1:1000 |
| p62 (HRP Linked) | Santa Cruz | sc-48402 HRP | WB | 1:1000 |
| Phospho-RelA  (p-Ser536) | Sigma Aldrich | SAB4300009 | WB | 1:1000 |
| RelA | Sigma Aldric | SAB4300295 | WB | 1:1000 |
| GAPDH (HRP Linked) | Cell Signaling Technologies | 8884 | WB | 1:1000 |
| β-Actin (HRP Linked) | Sigma Aldrich | A3854 | WB | 1:2000 |
| ECL Donkey anti-rabbit HRP Linked Whole Ab | Millipore Sigma | GENA934 | WB | 1:3000 |

**IHC** – immunohistochemistry; **WB** – Western Blotting
